# Volatile metabolomics and transcriptomics analyses provide insights into the mechanism of volatile changes during fruit development of ‘Ehime 38’ (*Citrus reticulata*) and its bud mutant

**DOI:** 10.3389/fpls.2024.1430204

**Published:** 2024-06-26

**Authors:** Jiaxian He, Zeyu Qin, Kexin Liu, Xiangyi Li, Yiming Kou, Zhenghua Jin, Ruiyuan He, Min Hong, Bo Xiong, Ling Liao, Guochao Sun, Siya He, Mingfei Zhang, Dong Liang, Xiulan Lv, Xun Wang, Zhihui Wang

**Affiliations:** ^1^ College of Horticulture, Sichuan Agricultural University, Chengdu, China; ^2^ Citrus Research Institute, Southwest University, Chongqing, China

**Keywords:** citrus, bud mutant, volatile compounds, fruit development, MEP pathway

## Abstract

Volatile compounds are important determinants affecting fruit flavor. Previous study has identified a bud mutant of ‘Ehime 38’ (*Citrus reticulata*) with different volatile profile. However, the volatile changes between WT and MT during fruit development and underlying mechanism remain elusive. In this study, a total of 35 volatile compounds were identified in the pulps of WT and MT at five developmental stages. Both varieties accumulated similar and the highest levels of volatiles at stage S1, and showed a downward trend as the fruit develops. However, the total volatile contents in the pulps of MT were 1.4–2.5 folds higher than those in WT at stages S2-S5, which was mainly due to the increase in the content of d-limonene. Transcriptomic and RT-qPCR analysis revealed that most genes in MEP pathway were positively correlated with the volatile contents, of which *DXS1* might mainly contribute to the elevated volatiles accumulation in MT by increasing the flux into the MEP pathway. Moreover, temporal expression analysis indicated that these MEP pathway genes functioned at different developmental stages. This study provided comprehensive volatile metabolomics and transcriptomics characterizations of a citrus mutant during fruit development, which is valuable for fruit flavor improvement in citrus.

## 1 Introduction

Citrus is one of the most economically valuable fruit crops worldwide and is well-loved by consumers because of its unique flavor and high nutritive value (González-Molina et al., 2010; Wang et al., 2022). Aroma is an important indicator of fruit flavor, and it is an attractive and distinguishing characteristic of citrus fruits (Pan X. et al., 2023; Zhang et al., 2023). More than 300 volatile compounds have been characterized in citrus, mainly including terpenoids, esters, alcohols, aldehydes, ketones, and acids (Perez-Cacho and Rouseff, 2008; Zhang et al., 2017). These volatile compounds not only facilitate plants to resist pathogen and herbivorous insects, but also play important roles in human health (Sharma et al., 2017; Anandakumar et al., 2021; Zhou et al., 2022).

Different composition and concentration of volatiles contribute to the distinctive aroma of fruits among citrus varieties. Monoterpenes are the most abundant volatiles in most citrus fruits, of which d-limonene, a component with a pleasant lemon scent which is widely used as an aroma additive in perfumes, cosmetics and foods, constitutes 60%-95% of the total volatiles content (Lota et al., 2001; Gonzalez-Mas et al., 2011; Zhang et al., 2017; Anandakumar et al., 2021). However, sesquiterpenes are dominantly (~55%) accumulated in the peels of *C. ichangensis* ‘Huaihua’, and trans-β-ocimene is the major monoterpene (Zhang et al., 2017). Valencene is a sesquiterpene with citrus-like scent that is mainly identified in sweet orange (Shen et al., 2016; Zhang et al., 2017), while citral is highly accumulated in lemon compared with sweet orange and pummelo, contributing to a strong lemon fragrance (Liu et al., 2012). In addition, the volatile profile is also significantly affected by developmental factors. It has been found that the content of limonene in orange peels shows a dramatic increase from approximately 60 days post anthesis (DPA) to 150 DPA and remains high until fruit fully ripens (Rodríguez et al., 2011). In the juices of mandarins, most monoterpenes tend to decrease as fruit matures, while aldehydes, alcohols, and esters increase (Hijaz et al., 2020).

As the most prominent volatiles in citrus, terpenoids are synthesized through two independent pathways: the MEP (2-C-methyl-D-erythritol-4-phosphate) pathway in plastids and the MVA (mevalonic acid) pathway in cytosol (Nagegowda, 2010; Liao et al., 2016; Volke et al., 2019). The MEP pathway is initiated with the condensation of pyruvate and G3P (glyceraldehyde-3-phosphate) catalyzed by DXS (1-Deoxy-D-xylulose-5-phosphate synthase) enzyme, and generates C5 units IPP (isopentenyl diphosphate) and DMAPP (dimethylallyl diphosphate) for the biosynthesis of GPP (geranyl pyrophosphate). The MVA pathway is started by enzyme AACT (AcAc-CoA thiolase), providing these two same C5 units for the synthesis of FPP (farnesyl pyrophosphate). Terpene synthases (TPSs) are responsible for the conversion of precursors GPP from MEP pathway and FPP from MVA pathway into monoterpenes (C10) and sesquiterpenes (C15), respectively. There are 55 predicted functional TPSs in sweet orange genome (Alquézar et al., 2017), and the functions of few genes have been characterized. Transient overexpression assays showed that *CitTPS16* catalyzes the synthesis of E-geraniol, contributing to flavor of sweet orange (Li X. et al., 2017). *CsTPS21* encodes a β-ocimene synthase responding to methyl jasmonic acid (MeJA) treatment, and overexpressing *CsTPS21* upregulates JA biosynthetic genes expression and increases the resistance to Asian citrus psyllid (Bin et al., 2023). However, little is known about the function of these genes and their associated pathways in aroma formation during citrus fruits development.

Bud mutations generate many new citrus cultivars, providing excellent materials for understanding the molecular mechanism underlying key traits formation. In the stay-green mutant ‘Green Ougan’, CrMYB68 retards the transformation of α-carotene and β-carotene via repressing the expressions of *CrBCH2* and *CrNCED5* (Zhu et al., 2017). The ‘Citrine Shiranui’ mandarin is a yellowish bud mutant with low levels of phytoene, β-cryptoxanthin, and 9-*cis-*violaxanthin, which may be due to the low expression levels of *PSY* and *ZDS* (Wang X. et al., 2023). The mutations of *STAYGREEN* (*SGR*), which simultaneously functions in carotenoid and chlorophyll accumulation, result in the brown appearance of navel orange mutant (Zhu et al., 2021). Pollen abortion is a vital agronomic trait for breeding engineering, and researchers have found that excessive starch and sugar hydrolysis caused by the demethylation of carbohydrate genes is correlated with the pollen development in a seedless citrus mutant (Ye et al., 2020). In a wax-deficient ‘Newhall’ orange mutant, the enhanced resistance to fungal infections is attributed to the increased biosynthesis of JA (He et al., 2018).

Metabolites are the end products of cellular processes in response to internal and external factors, and various advanced techniques have been developed for metabolomics profiling in horticultural fruits, including tomato (Zhu et al., 2018; Yang et al., 2024), sand pear (Shi et al., 2021), and apple (Dadwal et al., 2023). In citrus, high-performance liquid chromatography-mass spectrometry (HPLC-MS)-based widely targeted and non-targeted metabolomics platforms are employed to detect the primary and secondary metabolites (sugar, amino acid, flavonoid and other bioactive components) in different fruit tissues and different species (Wang et al., 2016; Dadwal et al., 2022; Wang S. et al., 2023), and gas chromatography-mass spectrometry (GC-MS) combined with headspace solid-phase micro-extraction (HS-SPME) is convenient and effective for aroma analysis, especially in fruit pulps (Hou et al., 2020; Li et al., 2022; Hu et al., 2024).

Our previous study has identified a yellow mutant of ‘Ehime 38’ (*Citrus reticulata*) with a richer aroma in pulps relative to the wild type (Wang T. et al., 2023). However, the accumulation patterns of volatile compounds during fruit development in both varieties and molecular mechanism remain elusive. In the present study, the composition and content of volatiles in the pulps of WT and MT across five developmental stages were detected by HS-SPME-GC-MS, and transcriptome analysis was used to understand the underlying mechanism of volatile variations between WT and MT. This study will provide new insights into the molecular mechanism of volatiles accumulation in citrus fruits.

## 2 Materials and methods

### 2.1 Plant materials

‘Ehime 38’ (*Citrus reticulata*) and its bud mutant, which are referred to as ‘WT’ and ‘MT’ respectively, were grafted onto different branches of the same rootstock ‘red tangerine’ (*Citrus reticulata*) in an orchard in Meishan city, Sichuan Province, China. Different developing fruits of WT and MT at 60, 90, 140, 170 and 210 days after full bloom (DAFB) were harvested, and fruit pulps were immediately separated and frozen in liquid nitrogen. Three biological replicates were performed for each sample, and one biological replicate contained three to ten fruits without mechanical injury and disease. All frozen pulps were stored at −80°C for further analysis.

### 2.2 Volatile compounds extraction and quantification

The extraction of volatiles was performed using solid phase micro extraction (SPME) according to previous study (Shen et al., 2016) with minor modifications. Frozen pulps were ground in liquid nitrogen and 0.5 g of powder was immediately transferred into a 20 mL headspace vial. The sample was incubated with 5 mL saturated sodium chloride solution and 50 μL 1-Hexanol (0.1%, v/v, used as an internal standard) at 42°C for 30 min. Then the SPME fiber coated with 50/30 µm divinylbenzene/carboxen/polydimethylsiloxane (Supelco, USA) was exposed to the headspace gases to extract the volatile compounds at 42°C for 30 min.

Volatile analysis was carried out on a gas chromatography (7890A, Agilent Technologies, Santa Clara) - mass spectrometry (Agilent 5975C) equipped with an HP-5MS column (30 m × 0.25 mm × 0.25 um, J&W Scientific, Folsom, CA, USA). The instrumental parameters were as follows: desorption in splitless mode at 250 °C for 5 min; oven temperature started at 40°C for 3 min, and gradually increased to 70°C at a rate of 3°C min^-1^, to 130°C at 1°C min^−1^, eventually to 230°C at 15°C min^−1^ and hod for 10 min; ion source, 230°C; electron energy, 70 eV; helium at 1.0 mL min^-1^, and mass scanning range 35–500 m/z. Data were processed by the enhanced MSD ChemStation software (Agilent MSD Productivity Chemstation). Volatile identification was performed using the NIST/EPA/NIH Mass Spectrometry Library (NIST-14, USA) combing with the Retention Indes (RI). A C8-C20 n-alkane mixed standard was analyzed under the same GC-MS condition to calculate the RI of volatiles. Volatile compounds were relatively quantified using the peak area of the internal standard (1-hexanol).

### 2.3 RNA isolation and transcriptomic sequencing

Total RNA of all samples was extracted according to the instructions of RNAprep Pure Plant Kit (Tiangen, China). Of them, developing fruits of WT and MT at 90 and 140 DAFB were chosen for RNA-seq analysis with three biological replicates and six fruits per replicate. The RNA-seq libraries were constructed using Hieff NGS Ultima Dual-mode mRNA Library Prep Kit for Illumina (Yeasen Biotechnology (Shanghai) Co., Ltd.), and sequenced on an Illumina NovaSeq platform at Biomarker Technologies (Beijing, China). After removing the low-quality reads and adapter sequence reads, clean reads were mapped to sweet orange reference genome (http://citrus.hzau.edu.cn/index.php) using Hisat2 tools soft (Kim et al., 2019). The FPKM (fragments per kilobase of transcript per million mapped reads) was used to represent genes expression level. Differentially expressed genes (DEGs) of pairwise samples were identified using the DESeq2 with a threshold of p value < 0.05 and | log2 (fold change) | ≥ 1.

### 2.4 Weighted gene co-expression network analysis

The R package WGCNA (v1.71) was used to analyze the co-expression networks of gene expressions and volatiles contents. The co-expression modules were constructed using an unsigned type of topological overlap matrix (TOM) with the soft thresholding power of 6, branch merge cut height of 0.15 and minimal module size of 100.

### 2.5 Quantitative real-time polymerase chain reaction analysis

The pulps of WT and MT at five developmental stages (60, 90, 140, 170, and 210 DAF) were used to extract total RNA. Each sample included three biological replicates. The extracted RNA (1 mg) was reacted with HiScript IV RT SuperMix (Vazyme Biotech) to synthesize single-strand cDNA for further analysis. Real-time PCR reactions were performed on a 96-well plate using the 2 × SYBR Premix EsTaq (Mei5 Biotechnology Co. Ltd, China). The temperature program in a CFX96 instrument (Hercules, CA, USA) was as follows: 95°C for 2 min, 40 cycles at 95°C for 10 s, and 55°C for 30 s. The 2^-ΔΔCt^ method was adopted to calculate gene relative expression level and citrus *actin* gene (ID: Cs1g_pb000860) was used for reference. The gene-specific primers were provided in **Supplementary Table S1**.

### 2.6 Statistical analysis

Orthogonal partial least squares-discriminant analysis (OPLS-DA) was used to identify differentially accumulated volatiles (DAVs) between WT and MT during fruit development. Principal component analysis (PCA) was performed by R package factoextra. The significant difference between two samples was calculated using the two-tailed paired t-test (*, p < 0.05; **, p < 0.01).

## 3 Results

### 3.1 Identification of volatiles in the pulps of WT and MT during fruit development

**Figure 1:**
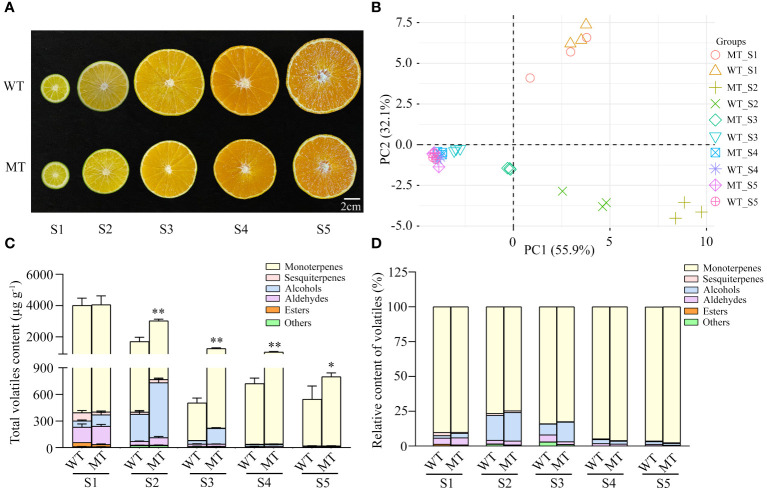
Phenotypes and total volatiles content in developing fruits of ‘Ehime 38’ (WT) and its bud mutant (MT). **(A)** Images of WT and MT at various developmental stages. Five stages from S1 to S5 refer to 60, 90, 140, 170, and 210 days after full bloom (DAFB), respectively. Bars = 2 cm. **(B)** PCA score of volatile profiles in the pulps of WT and MT during fruit development. **(C)** Total volatiles content in the pulps of WT and MT during fruit development. **(D)** Relative content of volatiles in the pulps of WT and MT during fruit development. Data were obtained from three biological replicates. Asterisks indicate statistically significant differences (two-tailed paired t-test, **p < 0.01; *p < 0.05).

To comprehensively investigate the volatile changes between WT and MT, different developing fruits were subjected to aroma profile analysis. Although MT was clearly distinguished from the orange-colored WT at ripening stages by its yellowish flavedo (Wang T. et al., 2023), there was no visible difference in pulp color between the two varieties during fruit development (**Figure 1A**). HS-SPME-GC-MS was adopted to evaluate the differences in volatiles composition and content of pulps between WT and MT. A total of 35 volatiles, including 12 monoterpenes, 7 aldehydes, 5 alcohols, 5 ketones, 3 sesquiterpenes, 2 esters, and 1 other component were detected simultaneously in both varieties (**Supplementary Table S2**). Principal component analysis (PCA) was performed to compare the volatile profile of WT and MT at different growth stages. The first two components explained 88% of the total variability (PC1 = 55.9% and PC2 = 32.1%). In the score plot, the developing fruits from S1 to S4 stage were clearly separated, while the pulps between stages S4 and S5 showed no significant difference. Notably, MT was obviously differentiated from WT at stages S2 and S3 (**Figure 1B**).

Similar accumulation patterns of total volatiles were present in the pulps of WT and MT. Both varieties accumulated the highest content of approximately 4000 µg g^−1^ at early stage (S1) and showed a downward trend with fruit development (**Figure 1C**). In addition, monoterpenes as the most abundant components firstly decreased from 91% to 73% of the total volatiles from stage S1 to S2, and then gradually increased to a maximum of 98% in fully mature fruits (S5) of WT and MT (**Figure 1D**). It was noteworthy that MT showed a slow decreasing rate of total volatiles, and its content was 1.4 to 2.5 folds higher than WT at S2 to S5 stages (**Figure 1C**). These results indicated that the volatile profiles dynamically changed during fruit development, and MT showed significant differences in volatile profiles compared to WT.

### 3.2 Analysis of differentially accumulated volatiles between WT and MT during fruit development

**Figure 2:**
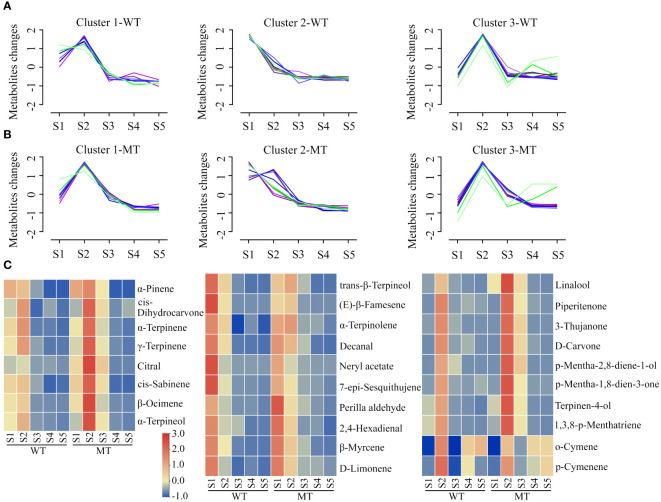
Analysis of differentially accumulated volatiles (DAVs) between WT and MT during fruit development. **(A)** Temporal accumulation patterns of DAVs in developing WT fruits. A total of 28 DAVs were grouped into three clusters by Mfuzz R package. **(B)** Accumulation trends of DAVs in developing MT fruits following the same clusters in WT. **(C)** Heatmaps of DAVs contents in the pulps of WT and MT during fruit development. The contents of DAVs were processed by in-row normalization, and a common scale was used for all heatmaps. These three heatmaps corresponded one-to-one with the Mfuzz clusters.

To further reveal the volatiles difference between WT and MT during fruit development, orthogonal partial least-squares discrimination analysis (OPLS-DA) was performed. Among these comparisons, the OPLS-DA models of three groups (MT_S1 *vs* WT_S1, MT_S2 *vs* WT_S2 and MT_S3 *vs* WT_S3) generated values above 0.75 for both R2 and Q2, and their permutation test illustrated that the Y-permuted Q2 values were lower than the original Q2 points and that the Q2 regression line had a negative intercept (**Supplementary Figure S1**), indicating MT was clearly distinguished from WT at the early (S1 stage) and middle stages (S2 and S3 stages) of fruit development. Based on the thresholds of variable importance in project (VIP) > 1, p value < 0.05, and fold change (FC) ≥ 2 or ≤ 0.5, a total of 28 differentially accumulated volatiles (DAVs) were screened (**Supplementary Table S3**). These DAVs were grouped into three clusters by Mfuzz according to their accumulation trends in WT (**Figure 2A**). Meanwhile, the volatiles in developing MT fruits were normalized and present in accordance with the same clusters in WT (**Figure 2B**). The results indicated that some DAVs exhibited different accumulation trends between WT and MT during fruit development.

The relative change of DAVs between WT and MT was visualized by heatmaps (**Figure 2C**), which displayed the corresponding volatiles in the three clusters. Almost all DAVs in cluster 1, except for α-pinene, showed the highest content at stage S2 in both WT and MT. These DAVs mainly accumulated at first two stages in WT, but highly accumulated at first three stages in MT. The contents of 8 DAVs (cis-sabinene, α-terpinene, α-pinene, β-ocimene, γ-terpinene, α-terpineol, cis-dihydrocarvone and citral) were higher in MT compared with WT at stages S2 and S3. Among them, citral was a low abundance monoterpene aldehyde and showed a relatively small change in WT during fruit development. The citral content in MT was 5.8- and 7.2- fold higher than that in WT at stages S2 and S3, respectively (**Supplementary Table S3**). For cluster 2, all volatiles in WT showed a downward trend as the fruit develops, while α-terpinolene, trans-β-terpineol and (E)-β-famesene increased at first and then decreased in MT. D-limonene was the most abundant volatile, accounting for 61%-87% of the total volatile contents, and its content in MT was higher (1.5–2.4 folds) than that in WT during S2-S5 stages. A similar accumulation trend to d-limonene was found for β-myrcene (the second highest volatile at S1 stage, 3.8%) and 2,4-hexadienal. In contrast to perilla aldehyde, four DAVs (trans-β-terpineol, (E)-β-famesene, neryl acetate and 7-epi-sesquithujene) showed notably lower contents (< 0.6-fold) in MT than those in WT at stage S1 (**Figure 2C**). Cluster 3 contained two groups: Group 1, consisting of 8 DAVs (linalool, 1,3,8-p-menthatriene, 3-thujanone, p-mentha-2,8-diene-1-ol, terpinen-4-ol, d-carvone, p-mentha-1,8-dien-3-one, piperitenone) mainly accumulated at S2 and/or S3 stages, and their abundance in WT was less in comparison with MT at these two developmental stages; Group 2, consisting of o-cymene and p-cymenene, primarily accumulated at S2, S4 and S5 stages. Among them, linalool amount was only significantly lower to that of d-limonene and comprised 5.3%-13.1% of the total volatiles content at S2 and S3 stages, while o-cymene had the second highest content with 7.0%-18.2% at S4 and S5 stages. In addition, linalool as the richest component among monoterpene alcohols showed greater than 2-fold difference between WT and MT during the early and middle stages of fruit development (**Supplementary Table S3**).

### 3.3 Transcriptomic analysis of developing fruit pulps of WT and MT

Given the large difference of volatile profile between WT and MT at stages S2 and S3 (**Supplementary Figure S1**), transcriptome sequencing analysis was carried on the fruit pulps of the two varieties at these two stages. After removing the low-quality reads and adaptor sequence, a total of 268.7 million clean reads with Q30 scores greater than 91.99% were obtained (**Supplementary Table S4**). These reads were subsequently mapped to the reference genome of sweet orange (Wang et al., 2021) with the average alignment rates of 91.08% (**Supplementary Table S5**). The correlations of 15 genes expression between qRT-PCR results and transcriptomic data were generally high (**Supplementary Figure S2**), indicating that the transcriptome sequencing data were reliable. Pairwise comparisons of transcriptomes with 4 groups were performed. The number of differentially expressed genes (DEGs) was largest in MT_S2 *vs* MT_S3 group with 2,371 upregulated and 2,802 downregulated genes, closely followed by 4,453 DEGs (2142 upregulations and 2311 downregulations) observed in WT_S2 *vs* WT_S3 group (**Supplementary Figure S4**). Only 688 and 699 DEGs were found in MT_S2 *vs* WT_S2 and MT_S3 *vs* WT_S3 comparison groups, respectively (**Supplementary Figure S3**).

**Figure 3:**
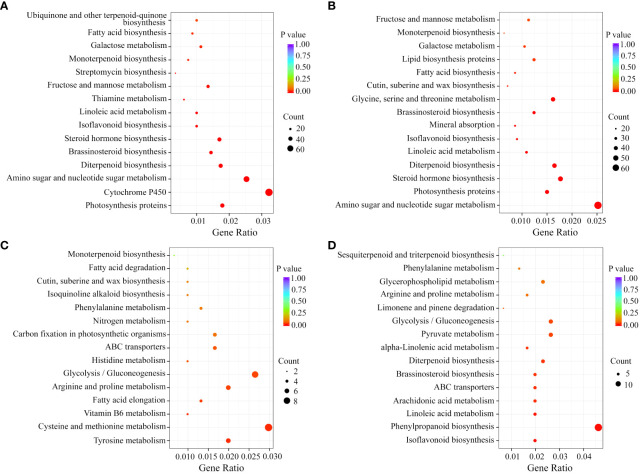
KEGG enrichment analysis of the DEGs in each comparison group. KEGG pathways of in comparison of WT_S3 and WT_S3 **(A)**, MT_S3 and MT_S3 **(B)**, MT_S2 and WT_S2 **(C)** and MT_S3 and WT_S3 **(D)**.

Kyoto Encyclopedia of Genes and Genomes (KEGG) enrichment analysis was performed on these DEGs. In the groups comparing the developing fruits from WT or MT, the DEGs were individually significantly enriched into 52 and 57 KEGG pathways with 36 common pathways. In addition to the fruit growth and development-related metabolism processes, such as ‘Amino sugar and nucleotide sugar metabolism’, ‘Photosynthesis proteins’, ‘Steroid hormone biosynthesis’, and ‘Fructose and mannose metabolism’, the 36 common pathways also included ‘Monoterpenoid biosynthesis’, ‘Linoleic acid metabolism’, and ‘Diterpenoid biosynthesis’ that were associated with volatile compounds synthesis (**Figures 3A, B**). In the MT_S2 *vs* WT_S2 comparison group, the most significantly enriched pathways were ‘Tyrosine metabolism’, ‘Cysteine and methionine metabolism’ and ‘Vitamin B6 metabolism’ (**Figure 3C**). In the MT_S3 *vs* WT_S3 comparison group, the enriched pathways included ‘Isoflavonoid biosynthesis’, ‘Linoleic acid metabolism’, ‘ABC transporters’, ‘Diterpenoid biosynthesis’, and ‘Limonene and pinene degradation’ (**Figure 3D**). These KEGG enrichment results indicated that monoterpenoid metabolism, linoleic acid metabolism and isoflavonoid biosynthesis might play important roles in modulating the volatile profiles of WT and MT during fruit development.

### 3.4 Identification of genes correlated with volatiles accumulation

**Figure 4:**
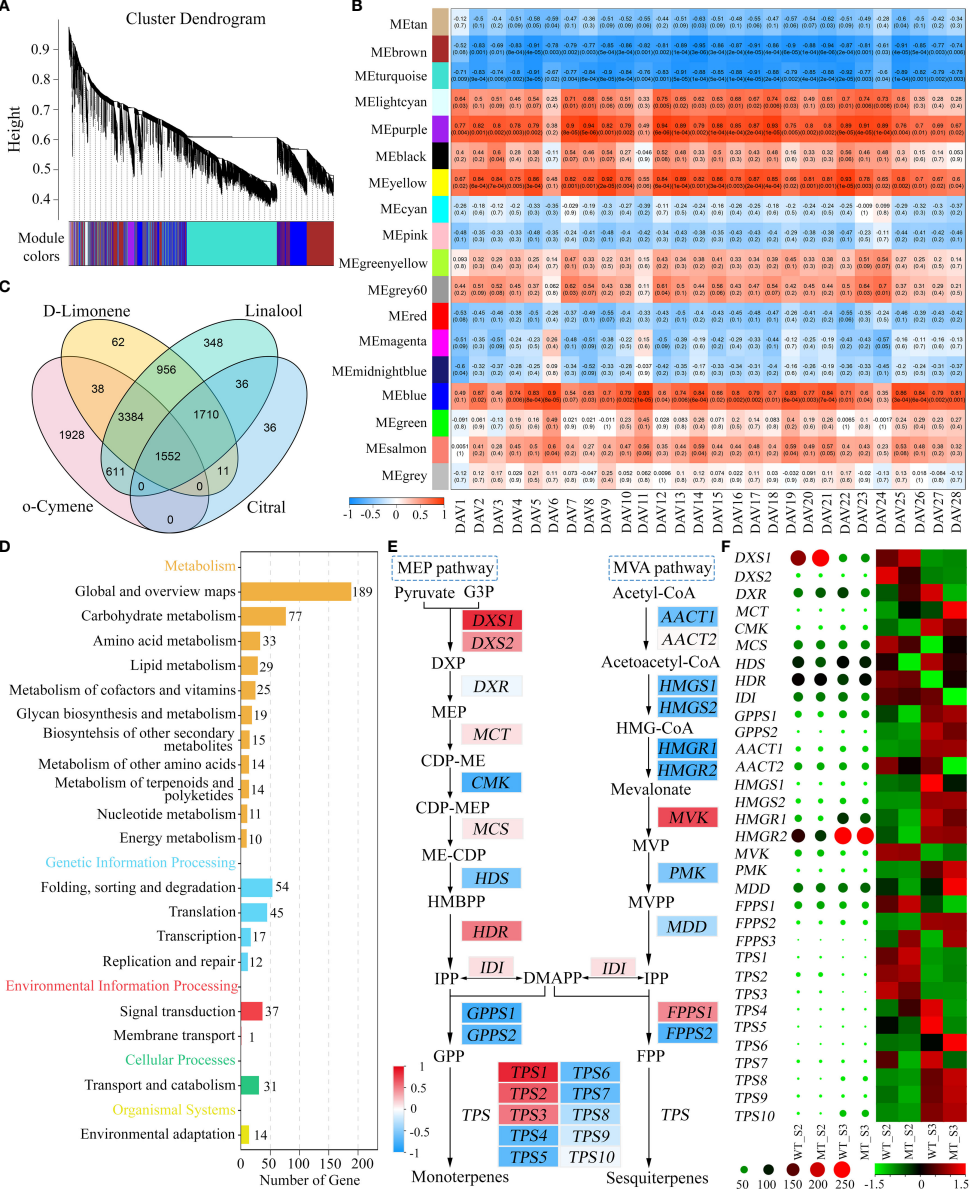
Identification of genes associated with DAVs accumulation in WT and MT. **(A)** Hierarchical clustering tree showing co-expression modules identified by WGCNA. **(B)** Correlations between DAVs and modules. The numbers in parentheses represent the significance, and the numbers above represent the correlation coefficient. **(C)** Venn diagram of candidate genes shared by d-limonene, linalool, o-cymene and citral. These genes were screened out from the turquoise, brown, purple, yellow, and blue modules of the four DAVs. **(D)** KEGG enrichment analysis of the common candidate genes. **(E)** Correlations of MEP and MVA pathway genes with d-limonene. D-limonene constituted the largest portion of total volatiles in both varieties, and it was chosen for representative of these DAVs. The background colors of genes represent the strength of the correlation. DXS, 1-deoxy-D-xylulose-5-phosphate synthase; DXR, 1-deoxy-D-xylulose-5-phosphate reductoisomerase; MCT, 2-C-methyl-D-erythritol 4-phosphate cytidylyltransferase; CMK, 4-(cytidine 50-diphospho)-2-C-methyl-D-erythritol kinase; MCS, 2-C-methyl-D-erythritol 2,4- cyclodiphosphate synthase; HDS, **(E)**-4-hydroxy-3-methylbut-2-enyl diphosphate synthase; HDR, **(E)**-4-hydroxy-3-methylbut-2-enyl diphosphate reductase; IDI, isopentenyl diphosphate isomerase; GPPS, geranyl diphosphate synthase; AACT, acetoacetyl-CoA thiolase; HMGS, 3-hydroxy-3-methylglutaryl-CoA synthase; HMGR, 3-hydroxy-3-methylglutaryl-CoA reductase; MVK, mevalonate kinase; PMK, phosphomevalonate kinase; MDD, mevalonate diphosphate decarboxylase; FPPS, farnesyl diphosphate synthase; TPS, terpene synthase. **(F)** Heatmaps of MEP and MVA pathway genes. The heatmap shown with circles was drawn based on the raw FPKM values, and that shown with rectangles was drawn based on the row-scaled FPKM values.

To explore the candidate genes involved in volatiles accumulation in WT and MT during fruit development, weighted gene co-expression network analysis (WGCNA) was performed to assess the correlations of genes and DAVs. After quality control, 17,344 genes were grouped into 18 modules (**Figure 4A**), which were marked with different colors. Among them, the turquoise module had the maximum number of genes (5,716), and the grey60 module had the lowest (114). It was shown that the turquoise and brown modules strongly negatively correlated with most of DAVs, while other three modules (purple, yellow, and blue modules) showed high positive correlations (**Figure 4B**). Given the similar results of correlation analysis of these DAVs, four representative DAVs (D-limonene, linalool, o-cymene and citral) were selected for screening candidate genes responsible for volatiles accumulation. There were 1,552 genes simultaneously well-correlated with the four volatiles (| r | > 0.60, p < 0.05) (**Figure 4C**). KEGG enrichment analysis of these common genes showed that they were mainly annotated in ‘Metabolism’ and ‘Genetic information processing’ pathways (**Figure 4D**). Of them, the pathway ‘Metabolism of terpenoids and polyketides’ was of particular interest, which included *DXS*, *CMK* (*4-(cytidine 50-diphospho)-2-C-methyl-D-erythritol kinase*), *HMGR* (*3-hydroxy-3-methylglutaryl-CoA reductase*), and *TPS* genes involved in MEP pathway and MVA pathway. In addition, these structural genes also showed high correlations (| r | > 0.70) with 21 out of the remaining 24 DAVs (**Supplementary Table S6**), indicating their importance in volatiles accumulation in WT and MT.

Monoterpenes were the most abundant volatiles in the pulps of WT and MT during fruit development (**Figure 1D**), which were synthesized from the product (GPP) of MEP pathway (Nagegowda, 2010). Most of MEP pathway genes positively correlated with these DAVs (**Supplementary Table S7**), of which d-limonene was selected for presentation (**Figure 4E**). Among them, *DXS* that encoded the first enzyme controlling the flux into MEP pathway exhibited strong positive correlations. In contrast, the genes involved in MVA pathway that produce sesquiterpenes generally showed negative correlations (**Figure 4E**). Terpene synthases (TPS) catalyzed the formation of monoterpenes and sesquiterpenes from GPP and FPP, respectively, and there were 3 *TPSs* positively related and 7 *TPSs* negatively related. Interestingly, heatmap analysis of raw or scaled FPKM values showed that *DXS1* expressed at a substantially high level at stage S2, and its expression level in MT was significantly higher than that in WT (**Figure 4F**). In addition, the expression of *HDR* in MEP pathway also showed a decreasing trend from stage S2 to S3, while the expression of *HDS* significantly increased during the two periods. The genes in MVA pathway with high FPKM values such as *HMGR1*, *HMGR2* and *MDD* (*mevalonate diphosphate decarboxylase*) exhibited inconsistent trends with these DAVs (**Figure 4F**). These results indicated that the MEP pathway, especially *DXS1* gene, played important and positive roles in volatiles accumulation in WT and MT at stages S2 and S3.

### 3.5 Analysis of the critical genes related to volatiles metabolism in WT and MT throughout fruit development

**Figure 5:**
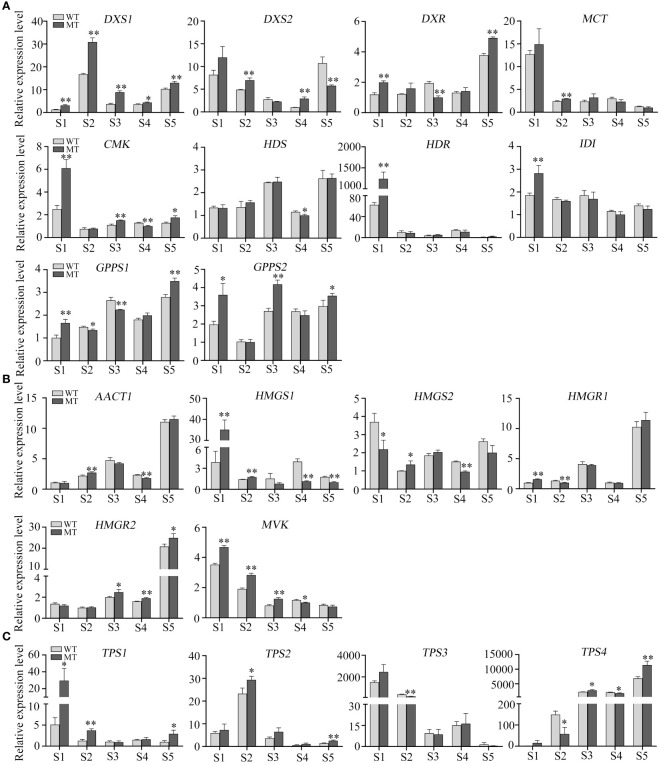
Relative expression levels of critical genes in WT and MT across fruit development. **(A)** Relative expression of MEP pathway genes in the pulps of WT and MT across fruit development. **(B)** Relative expression of MVA pathway genes. **(C)** Relative expression of *TPSs*. Data are expressed as means + SD, n = 3. Statistical analysis: Two-tailed paired t -test; *p < 0.05; **p < 0.01.

To further evaluate the roles of MEP and MVA pathways in volatiles accumulation, the expression levels of structural genes in WT and MT across fruit development were analyzed. In MEP pathway, *DXS1* was consistently expressed at higher levels in MT than that in WT during fruit development (**Figure 5A**), indicating its critical role in determining the differential accumulation of volatiles between WT and MT. However, *DXS1* showed a very low expression level at S1, although this period had the highest total volatiles. Investigation of pathway genes indicated that the expression levels of *MCT*, *CMK*, and *HDR* were much higher at S1 than those at other four stages (**Figure 5A**). Comparing genes’ expression between the two varieties, we found that *DXS1*, *DXS2*, and *MCT* expressed at higher levels in MT than WT at S2, and *DXS1*, *CMK*, and *GPPS2* expressed at higher levels in MT than WT at S3 (**Figure 5A**). These DEGs may be relevant to the variations of volatiles between WT and MT at S2 and S3 stages. In MVA pathway, the expression of *AACT1*, *HMGR1*, and *HMGR2* increased first, and then decreased and then increased, reaching the maximum at stage S5 (**Figure 5B**). The opposite was seen with *MVK*, it had the highest expression at S1 and showed a decreasing trend along fruit development (**Figure 5B**). In addition, *MVK* significantly differentially expressed between WT and MT. The results indicated that *MVK* might play important role in determining the accumulation of sesquiterpenes including 7-epi-sesquithujene and (E)-β-famesene in WT and MT.

In addition, we also analyzed the expression pattern of four *TPS* genes. All *TPS1*, *TPS2*, and *TPS3* positively correlated with the DAVs (**Figure 4F**), of which *TPS1* and *TPS3* was expressed the highest at stage S1, and *TPS2* exhibited the highest level at S2 (**Figure 5C**). Their expression trends were consistent with those observed in volatiles, as almost all DAVs showed the highest amount at stage S1 or S2 (**Figure 2A**). *TPS4* was the most negatively correlated with the DAVs (**Figure 4F**) and its expression level showed a clear upward trend as fruit develops (**Figure 5C**), thus this gene might be associated with the accumulation of the volatiles, such as valencene and o-cymene, which were highly accumulated at late stages.

## 4 Discussion

Volatile compounds not only are the important determinants of fruit quality perceived by consumers, but also are beneficial to human health (Liu et al., 2021; Pan X. et al., 2023). Citrus fruit has a strong aroma which is altered by developmental factors, thus analysis of volatile changes of citrus mutant during fruit development helps to better understand the mechanism of volatiles accumulation.

The volatile profiles of pulps in WT and MT at five developmental stages were determined. The composition of volatiles showed no difference between two varieties, and a total of 35 volatile compounds were detected in both varieties across fruit development, including terpenes, aldehydes, alcohols, ketones and esters. For total volatiles content, MT had significantly higher contents of total volatiles than WT at the stages S2-S5, although both of them were most abundant at stage S1 with a similar level. To better understand the difference between WT and MT, we performed OPLS-DA to identify differentially accumulated volatiles (DAVs) and analyzed their accumulation patterns during fruit development. Among these 28 DAVs, d-limonene was always the most dominant volatile throughout fruit development, which was similar to previous researches (Hou et al., 2020; Hu et al., 2024), and its average content in MT was 1.5–2.4 folds higher compared with WT during S2-S5 stages. Linalool was the richest component among monoterpene alcohols associated with typical sweet floral scent, exhibiting significant higher levels (2.3–4.4 folds) in MT than that in WT at S1-S3 stages. Other DAVs such as trans-β-terpineol, (E)-β-famesene, and α-terpinolene also showed different accumulation patterns between WT and MT. In addition to the differences between two varieties, there were also large changes in volatiles composition and content during fruit development. Valencene was specifically accumulated at the final two periods as previously reported (Shen et al., 2016; Hou et al., 2020), and octyl acetate was detected only in the first one period. In our study, the content of d-limonene in pulps decreased as the fruit develops, which was inconsistent with previous data (Hou et al., 2020; Kim et al., 2022). This may be due to that different citrus varieties were measured, or that our sampling time was earlier compared with previous studies. The latter speculation was supported by that the content of monoterpenes was much higher in the pulps of a very early-ripening pummelo ‘LYZ’ than that in other three pummelos (Pan T. et al., 2023).

Plant monoterpenes and sesquiterpenes are generally produced by the plastidial MEP pathway and the cytosolic MVA pathway, respectively (Nagegowda, 2010). It has been reported that the high expression levels of five genes in MVA pathway are associated with the high content of sesquiterpenes in the leaves of wild or semiwild citrus germplasms (Zhang et al., 2020). Monoterpenes constituted the largest portion of total volatiles in both WT and MT, and the expression of most genes in MEP pathway were positively correlated with the level of most DAVs (including d-limonene) and most genes in MVA pathway were negatively correlated. DXS is the first key rate-limiting enzyme in MEP pathway, and overexpressing *DXS* significantly enhances monoterpenes content in *Nicotiana benthamiana* (Nieuwenhuizen et al., 2015; Tian et al., 2022). In the pulps of MT, *DXS1* was consistently expressed at higher levels than that in WT during fruit development. Moreover, the higher expression levels of *DXS2*, *MCT*, *CMK*, and *GPPS2* in MT than those in WT might also contribute to the large variations between two varieties at S2 and S3 stages. A previous study indicated that the decreased volatiles contents were closely related to the increased carotenoids contents in a red-flesh ‘HJH’ pummelo mutant (Zhu et al., 2022). In other citrus red-flesh mutants such as ‘R-An’, ‘R-GX’ and ‘NRH’, the total volatiles and carotenoids contents increased, and the ABA and/or limonoid aglycones contents decreased (Li W. et al., 2017; Liu et al., 2019). These researchers have proposed that the varied metabolites profiles are due to the flux balance of terpenoid metabolism rather than the increased flux into the isoprenoid pathway (Li W. et al., 2017; Liu et al., 2019; Zhu et al., 2022). In this study, MT had significantly higher expression levels of *DXS1* than WT, and the elevated volatiles accumulation in MT could be attributed mainly to the increased flux into MEP pathway. In addition, we found that *DXS2*, *MCT*, *CMK*, and *HDR* were active at S1, and *DXS1* was active at S2-S5 stages. These results indicated that different MEP pathway genes functioned at different stages of fruit development in WT and MT.

*TPSs* are key genes for terpene biosynthesis, few of which have been functionally characterized in citrus (Shen et al., 2016; Li X. et al., 2017; Bin et al., 2023). There were 3 *TPSs* and 7 *TPSs* positively and negatively correlated with the DAVs, respectively. Among them, *TPS4* was the most strongly negatively correlated with the DAVs. It has been reported that TPS4/VS mainly catalyzes the formation of valencene in navel orange (Hu et al., 2024). qRT-PCR assays showed that the expression of *TPS4* was increased gradually during fruit development in WT and MT, which was consistent with the accumulation pattern of valencene. Moreover, the expression trends of *TPS1*, *TPS2* and *TPS3* were similar to those observed in the DAVs during fruit development, and they were significantly differentially expressed between WT and MT at stage S2. These results indicated the important and positive roles of the three *TPSs* in volatiles accumulation of WT and MT, and this needs further functional validation.

## 5 Conclusions

A total of 35 volatile compounds were identified in the pulps of WT and MT during fruit development by HS-SPME-GC-MS, and differences in volatile profiles between both varieties at five developmental stages were analyzed by OPLS-DA. Compared with WT, the contents of total volatiles were significantly higher in MT at stages S2-S5, of which d-limonene was the most predominant volatile. Transcriptomic and qRT-PCR analysis revealed that the genes in MEP pathway, especially *DXS1*, played important and positive roles in the differential accumulation of volatiles accumulation in WT and MT during fruit development. These results could provide insights into the aroma characteristics of developing citrus fruit and the molecular mechanism underlying volatiles accumulation, which is of great importance in improving fruit flavor in citrus.

## Data availability statement

The datasets presented in this study can be found in online repositories. The RNA-seq datasets can be found at: https://www.ncbi.nlm.nih.gov/, PRJNA1124587.

## Author contributions

JH: Conceptualization, Data curation, Formal analysis, Funding acquisition, Methodology, Writing – original draft. ZQ: Conceptualization, Data curation, Formal analysis, Methodology, Resources, Writing – original draft. KL: Data curation, Methodology, Writing – original draft. XYL: Methodology, Writing – review & editing. YK: Methodology, Writing – review & editing. ZJ: Resources, Writing – review & editing. RH: Methodology, Writing – review & editing. MH: Methodology, Writing – review & editing. BX: Project administration, Resources, Writing – review & editing. LL: Project administration, Writing – original draft. GS: Project administration, Resources, Writing – original draft. SH: Project administration, Writing – original draft. MZ: Formal analysis, Project administration, Writing – review & editing. DL: Supervision, Writing – review & editing. XLL: Supervision, Writing – review & editing. XW: Supervision, Writing – review & editing. ZW: Funding acquisition, Project administration, Supervision, Writing – review & editing.
