# Supplementary material for: Volatile metabolomics and transcriptomics analyses provide insights into the mechanism of volatile changes during fruit development of ‘Ehime 38’ (Citrus reticulata) and its bud mutant
Source: Front Plant Sci. 2024 Jun 26;15:1430204. doi: 10.3389/fpls.2024.1430204 (PMC11231921; doi:10.3389/fpls.2024.1430204)
Supplement: Supplementary file 1 [file DataSheet_1.docx]

**Supplementary data**


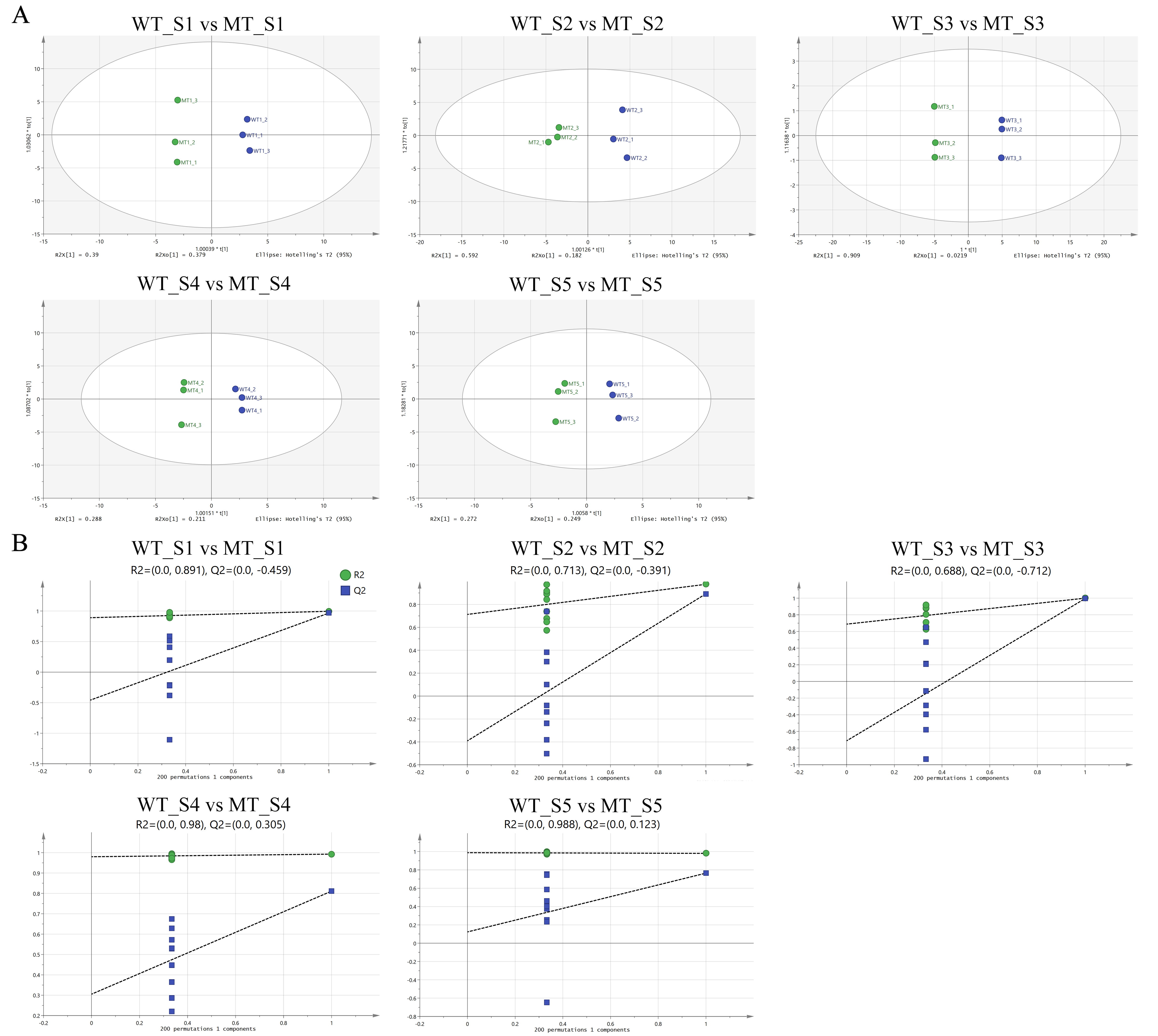


Supplementary Fig. 1. OPLS-DA analysis of the volatiles from WT and MT during fruit development. (A) OPLS-DA score plots of volatiles profile from WT and MT at the same developmental stage. (B) Permutation tests of these OPLS-DA models.


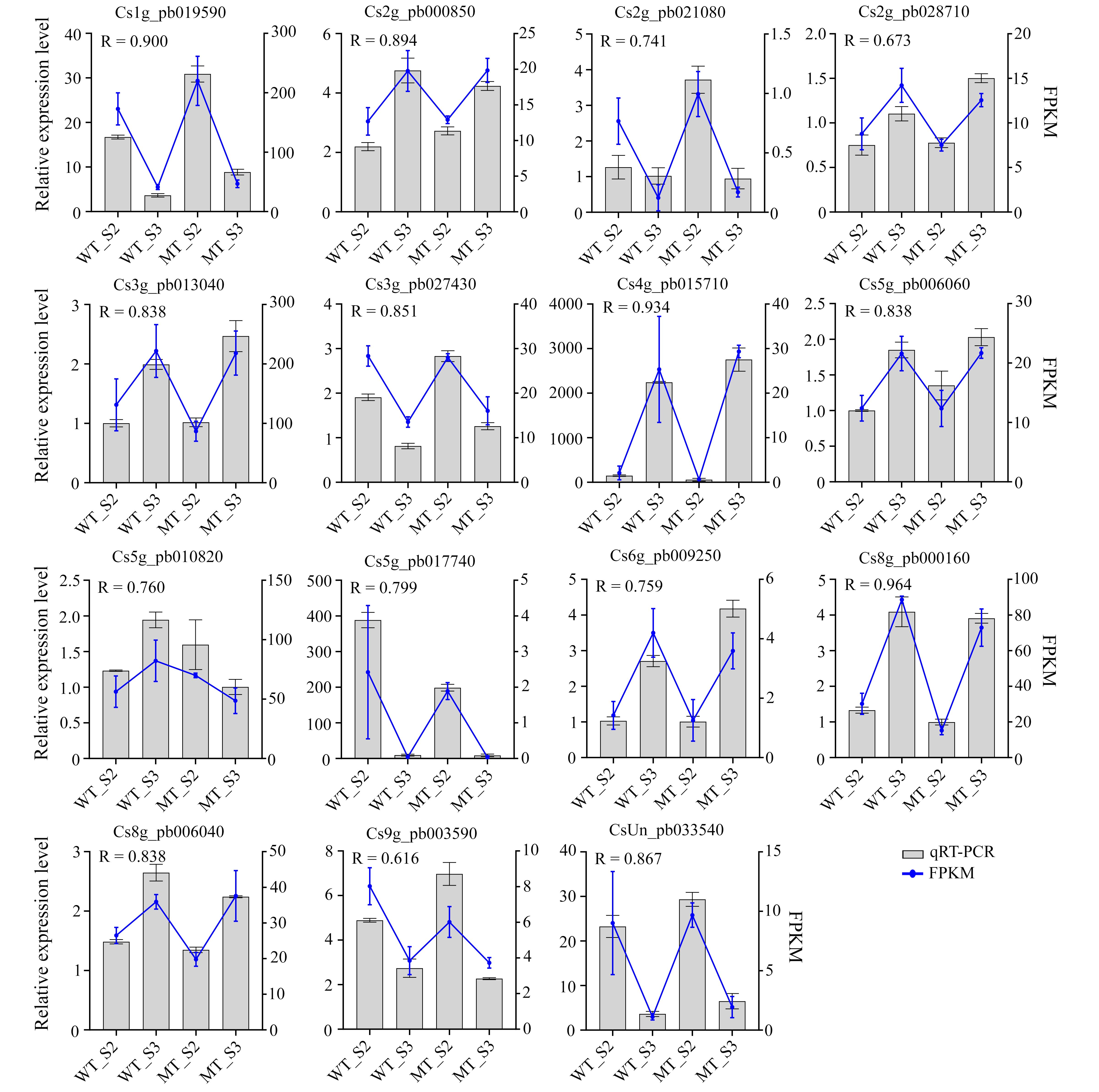


Supplementary Fig. 2. Correlation analysis of the transcriptomic data and qRT-PCR results. A total of 15 genes were analyzed, and their data were extracted from Fig. 5 for correlation analysis. The data bars represented means ± SD (n = 3). For each gene, Pearson’s correlation coefficient was calculated by comparing qRT-PCR and transcriptomic data.


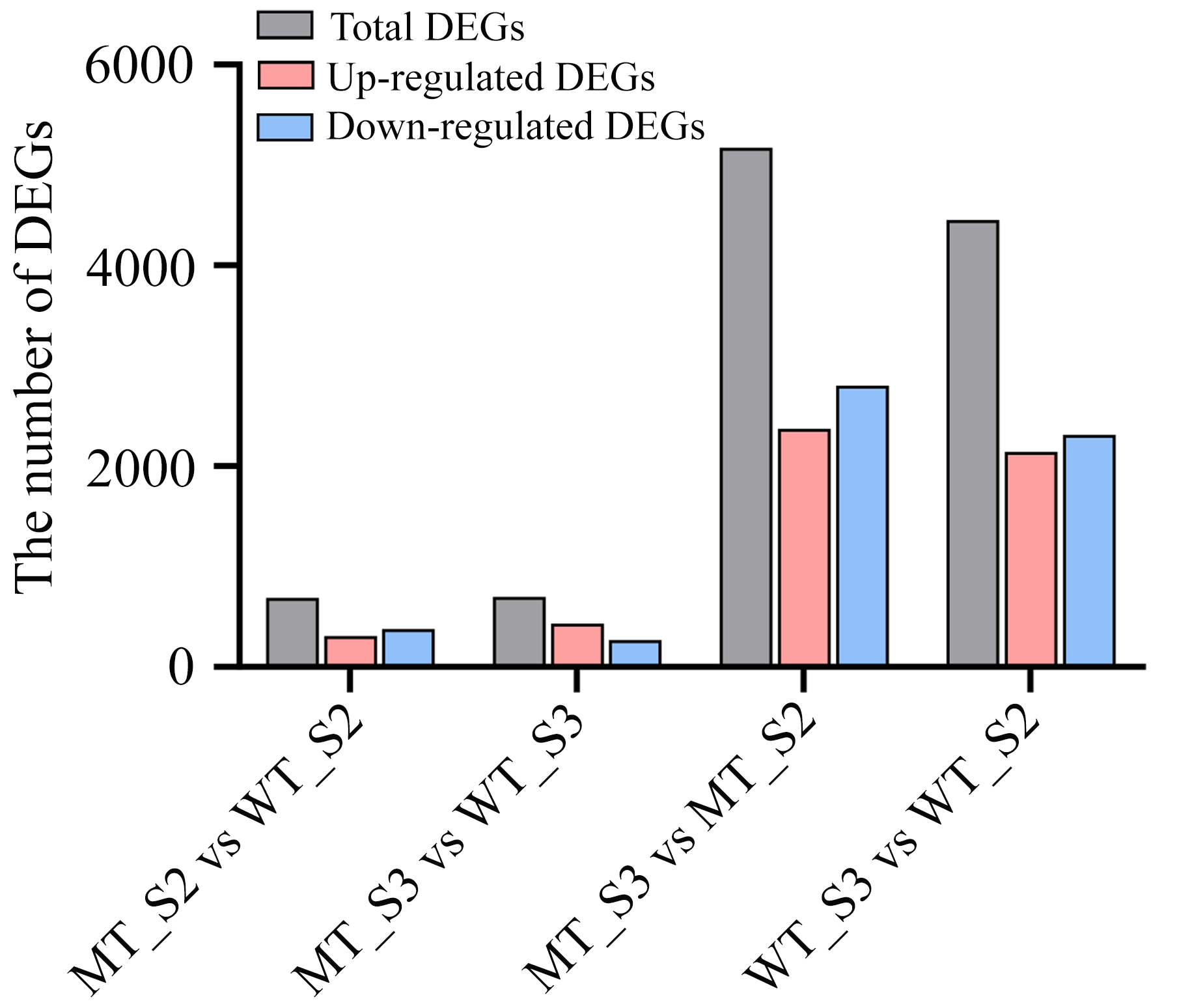


Supplementary Fig. 3. The number of DEGs of four comparison groups. Groups MT_S2 vs WT_S2 and MT_S3 vs WT_S3 were generated by comparing MT with WT at the same developmental stages, and Groups MT_S2 vs WT_S2 and MT_S3 vs WT_S3 were generated by comparing the developing fruits from WT or MT.
